# The role of Rho-associated kinase inhibitor, Y-27632 on primary culture of ovine spermatogonial stem cells

**DOI:** 10.1590/1984-3143-AR2020-0257

**Published:** 2022-01-07

**Authors:** Fatemeh Emamdoust, Mehdi Aminafshar, Mohammad Zandi, Mohammad Reza Sanjabi

**Affiliations:** 1 Department of Animal Science Faculty of Agriculture and Natural Resources, Science and Research Branch Islamic Azad University Tehran Iran Department of Animal Science, Faculty of Agriculture and Natural Resources, Science and Research Branch, Islamic Azad University, Tehran, Iran; 2 Department of Agriculture Iranian Research Organization for Science and Technology Tehran Iran Department of Agriculture, Iranian Research Organization for Science and Technology, Tehran, Iran

**Keywords:** spermatogonial stem cells, primary culture, Y-27632, sheep

## Abstract

The access to sufficient numbers of spermatogonial stem cells (SSCs) is a prerequisite for the study of their regulation and further biomanipulation. Rho kinase (ROCK) belongs to a family of serine/threonine kinases and involves in a wide range of fundamental cellular functions. The aim of the present study was to study the effect of ROCK inhibitor, Y-27632 (0.1-40 µM), during the primary culture of ovine SSCs. SSCs were collected from 3-5-month-old’s lamb testes. The viability of SSCs, the apoptosis assay of SSCs, the intracellular reactive oxygen species (ROS) analysis, and the SSCs markers and apoptosis-related gene expressions were detected by MTT reduction assay, Annexin V–FITC/ Propidium Iodide (PI) dual staining, flow cytometry and real-time-PCR studies, respectively. Morphological analyses indicated that the 5-10 µM Y-27632 had an optimal effect on the number of presumptive SSCs colonies and the area covered by them after a 10 days culture. The cell viability, apoptosis and necrosis of SSCs after 10 days’ culture were not affected in comparison with the control group, and the 20 µM of Y-27632 resulted in significantly decreased cell viability (P<0.05) and an increased necrosis of cells. On day 10 after culture, the expression of *P53* was decreased with an increase from 0 to 10 µM in the Y-27632 dose. In the 20 µM Y-27632 group, the expressions of *P53* and *Bax* were higher and the *Bcl-2* was lower than other groups and these values were significantly different from 5 and 10 µM Y-27632 groups (P<0.05). The level of intracellular ROS was decreased with an increase in the Y-27632 dose from 5 to 20 µM in comparison with the control group. In conclusion, the present study demonstrated that Y-27632 at a concentration of 5-10 µM provided optimal culture conditions for the primary culture of ovine SSCs.

## Introduction

Spermatogonial stem cells in the mammalian testis are unipotent stem cells, which demonstrate distinctive cell features as stem and germ cells after being separated from the testis and cultured in vitro (Sahare et al., 2018). Among the stem cells in an adult male body, SSCs are very unique (Heidari et al., 2012) since they can transmit genetic information from one generation to another, and it is of significant importance (Shams et al., 2017). The fact mentioned above makes it possible for in vitro culture of SSCs (Dobrinski, 2006). Studies on SSCs have been initiated a few decades ago, concentrating on the production of goat and sheep offspring (Binsila et al., 2018). Using this technology in sheep is of great importance due to the problems associated with cryopreservation of ram sperm and artificial insemination and embryo transfer in ewes (Rodriguez-Sosa et al., 2006).

Rock inhibitor (also known as Y-27632) plays an important role in cellular functions, such as cellular polarity, proliferation, migration, metabolism, differentiation, cytokinesis, and apoptosis (Riento & Ridley, 2003; Pirone et al., 2006; Pertz, 2010).

The serine/threonine effectors ROCK I and ROCK II mediate the downstream signaling of Rho activation. In addition, Y27632 (Trans4 [(1R) amino ethyl] N4pyridinylcyclohexanecarboxamidedihydrochloride) is a powerful and influential inhibitor of ROCK I and ROCK II. It has been demonstrated that the Y-27632, would inhibit apoptosis, further cell viability after seeding, and control cell differentiation in embryonic stem cells (Watanabe et al., 2007). Moreover, the survival of cryopreserved dissociated human embryonic stem cells (hESCs), human induced pluripotent stem cells (hiPSCs), and human mesenchymal stem cells would be promoted by this inhibitor (Heng, 2009). The survival of additional cells such as endothelial cells (Koga et al., 2006), retinal ganglion cells (Abbasi et al., 2013) and hESCs derived cardio myocyte and non-cardio myocyte cells (Emre et al., 2010), is enhanced through applying the Y-27632. However, further studies are still required to examine whether the treatment of ovine SSCs with Y-27632 would facilitate stem cells survival and colony formation.

This study aims at improving primary culture of SSCs by adding different concentrations of Y-27632 on viability, colony formation, proliferation and apoptosis of SSCs. The effectiveness of Y-27632 was evaluated by different cellular functions such as viability, colony formation, proliferation and apoptosis, which are important in SSCs maintenance.

## Materials and methods

### Chemicals

Unless otherwise mentioned, all the chemicals used in the present work were purchased from Sigma (St. Louis, MO, USA). In addition, the plastics were bought from Sorfa (China).

### Isolation and culture of SSCs

The protocol for animal use in the present research was approved by the Iranian Research Organization for Science and Technology (IROST) Agricultural Institute of Animal Ethics, Care and Use (approval number: 2019-2). Testes from three different old shal ram lambs at 27.1 ± 4.1 kg average weight were collected from the local slaughterhouse and were brought to the laboratory within 2h of slaughter. A modified form of the two-time enzymatic digestion process described by Izadyar et al. (2002) was employed in order to isolate SSCs. For the first enzymatic digestion, 2 grams of minced seminiferous tissue of one testicle were suspended in Dulbecco’s Modified Eagle Medium (DMEM) (Inoclon, Iran) containing 1 mg/mL trypsin (Inoclon), 1 mg/mL hyaluronidase type II, 1 mg/mL collagenase and 5 μg/mL DNase. The minced seminiferous tissue was incubated in a shaker incubator (200 cycles/min) at 37 ºC for 45 min. The dispersed tissue was collected and subjected to centrifugation at 30 g for 2 min. The supernatant was collected and the pellet was washed with DMEM. For the second enzymatic digestion, the pellet was suspended in DMEM containing 1 mg/mL hyaluronidase type II, 1 mg/mL collagenase and 5 μg/mL DNase without trypsin. The pellet was incubated in a shaker incubator (200 cycles/min) for 30 min. The suspension was then centrifuged at 30 g for 2 min and the pellet was re-suspended in DMEM medium and used for enrichment of SSCs.

### Enrichment of SSCs, preparation of feeder layers and culture of SSCs

As to the enrichment of SSCs, the suspension was filtered through an 80-µm and a 60-µm nylon net filter sequentially. The filtered cells were then transferred to lectin-bovine serum albumin (BSA) coated with 60-mm petri dishes. The lectin-BSA coated dishes were prepared by dissolving the lectin (5 µg/mL) from *Datura stramonium* agglutinin into Dulbecco’s Phosphate-Buffered Saline (DPBS). The dishes were kept for 2h at room temperature and were then washed with BSA (0.6% BSA in DPBS). After that, the dishes were kept at room temperature for another 2h for coating BSA. The cells seeded on the lectin-BSA coated dishes were incubated for 5-6h in a CO_2_ incubator with 5% CO_2_ in air at 37 °C. These cells were incubated in order to enable the sertoli cells to get attached to the lectin-BSA. Next, the remaining medium, which was expected to contain SSCs, was collected and transferred to a 15-mL tube. It was then centrifuged for 5 min at 30 g, following which the supernatant was discarded and the pellet was re-suspended in DMEM.

To prepare feeder layers, the cells left over in the lectin-BSA coated dishes were revitalized with fresh DMEM supplemented with 10% fetal bovine serum (FBS) (Gibco, Life Technologies, Rockville, MD, USA) and transferred to an incubator at 37° C and 5% CO_2_ for 2-3 days in order to enable these cells, which were expected to be primarily sertoli cells, to grow till a confluent monolayer was formed. For propagation, the cells were sub-cultured in a 50-ml cell culture flask after being disaggregated with 0.25% trypsin-EDTA. For a feeder layer preparation, sertoli cells were plated at a density of 6×10^4^ cells/cm2 cells/cm^2^ and inactivated by 10 μg/mL mitomycin-C treatment for 3h. The cells were subsequently washed 5 times with DPBS and with DMEM supplemented with 10% FBS.

To culture SSCs, the isolated SSCs were cultured (6×10^4^ cells/cm^2^) on the sertoli cells feeder layer in 50-ml cell culture flasks containing DMEM medium supplemented with 10% FBS and in the presence of 0.1, 1, 2.5, 5, 10 and 20 µM of Y-27632. The SSCs were then incubated in a CO_2_ incubator with 5% CO_2_ in atmosphere at 37 °C. After 10 days, SSCs colonies were observed in the primary culture as described by (Kala et al., 2012).

The SSCs were characterized at passage 0 (primary culture). Alkaline phosphatase (AP) staining and the expression of *Plzf*, *Cmyc*, *Vasa* and *Thy1* genes were used for characterization of SSCs. For AP staining, SSCs colonies were washed twice with DPBS and were then stained using an AP kit (Sigma, Catalogue No.86C) as per manufacturer’s protocol. Surface area of the colonies was measured with Image J software (National Institutes of Health).

### RNA isolation, reverse transcription and real-time-PCR

Total RNA was isolated with Trizol reagent (Invitrogen Corp., Carlsbad, California, USA) and was subsequently treated with DNase (Ambion Inc., Houston, Texas, USA) in order to avoid DNA contamination. RNAs (100 ng) were used to generate the first-strand cDNA by using Moloney murine leukemia virus (MMLV) enzyme and oligo dT primers (Takara, Japan). The changes in the expression of specific markers and apoptotic-related genes were studied by real-time-PCR (Magnetic Induction Cycler (Mic) PCR Machine - Bio Molecular Systems, Australia). PCR was set up in a final volume of 10 μL having 5 µl SYBR Green (Genaxxon, bio science), 1.4 µl nuclease-free water, 0.8 µl of each primer (forward and reverse), and 2 µl template. The real-time-PCR program was started with an initial melting cycle at 94 °C for 15 min to activate the polymerase. The process was followed by 40 amplification cycles of denaturation at 95 °C for 10 sec, and the annealing of specific primers at 60 °C for 15 sec and at 72 °C for a 20 sec extension. The reactions were ended with a final extension at 72 °C for 5 min.

The following custom primer sequences were used for real-time-PCR gene expression analysis: *β-actin* [5'ACCCAGCACGATGAAGATCA3' (forward) and 5'GTAACGCAGCTAACAGTCCG3' (reverse)] (Accession number: U39357.1) (Annealing temperature: 60 °C); *Plzf* [5'CCTCAGATGACAATGACACG3' (forward) and 5'CGCCTTGGTGGGACTCA 3' (reverse)] (Accession number: NM_001037476.2) (Annealing temperature: 60 °C); *Cmyc* [5' AGAATGACAAGAGGCGGACA 3' (forward) and 5' CAACTGTTCTCGCCTCTTC 3' (reverse)] (Accession number: NM_001009426.1) (Annealing temperature: 60 °C); *Vasa* [5' TCTTGGAGATTTCCGCTG 3' (forward) and 5' GGCTGTGCTAACTGGCTA 3' (reverse)] (Accession number: JX437186.1) (Annealing temperature: 60 °C); *Thy1* [5'CGTCTCCAATAAGGATGTC3' (forward) and 5'GTCACAAGGAGATGAAGTC3' (reverse)] (Accession number: NM_001034765.1) (Annealing temperature: 60 °C); *Bcl-2* [5' GATGACTTCTCTCGGCGCTA 3' (forward) and 5'GACCCCTCCGAACTCAAAGA3' (reverse)] (Accession number: AY547260.1) (Annealing temperature: 60 °C); *Bax* [5'GTGAGACCTCTAACCCCACC3' (forward) and 5'GGTCAGAGGTCATGAGGAGG3' (reverse)] (Accession number: GU731063.1) (Annealing temperature: 60 °C); p53 [5'ACAACCTTCTGTCCTCCGAG3' (forward) and 5'GTTGCCAGGGTAGGTCTTCT3' (reverse)] (Accession number: FJ855223.1) (Annealing temperature: 60 °C). Primer3 software was used to design primers. The data were analyzed using the comparative threshold cycle (CT) method with *β-actin* used as an endogenous control. Relative expression for each gene was calculated as a ratio between target gene expression and its reference by ∆∆CT analysis. Fold change of gene expression was calculated as a ratio of expression levels of treated groups to the expression level of the control group (Livak and Schmittgen, 2001). *Plzf, Cmyc, Vasa* and *Thy1* genes were used as SSCs Markers and *Bax, Bcl-2* and *P53* genes were used as apoptosis related genes. The specificity and integrity of PCR products was ensured through the melt curve analysis. No PCR products were obtained when reverse transcriptase was omitted from cDNA synthesis or when DNA templates were omitted from the PCR reaction.

### MTT (Methylthiazolyldiphenyl-tetrazolium bromide) reduction assay

The feeder layer was prepared by coating 96-well dishes with mytomicin treated sertoli cells. After one day, SSCs were seeded on the layer. After 48h of cell culturing with 0.1, 1, 2.5, 5, 10, 20 and 40 µM Y-27632 in 96-well dishes (5000 cells per well), the viability of cells was determined using a kit (Thermo Fisher Scientific, ROCKford, IL, USA) as per manufacturer’s protocol. Briefly, 12 mM MTT stock solution was prepared by adding 1 mL of sterile PBS to 5 mg of MTT. Then, 10 µL of the 12 mM MTT stock solution was added to each well, which included a negative control of 10 µL of the MTT stock solution added to 100 µL of medium alone, and incubated for 4h with 5% CO_2_ at 37 °C. After that, 100 µL of SDS-HCl solution (10 mL of 0.01 M HCl was added to 1 gr of SDS) was added to each well. The microplate was incubated overnight at 37 °C and in a humidified chamber. The formazan concentration was determined by optical density at 570 nm. For each treatment, a coated 96-well dishes without SSCs were used to remove the effect of sertoli cells.

### Cell apoptosis assay

The FITC Annexin V Apoptosis Detection Kit with PI kit (BioLegend, UK) was used to detect the apoptosis of cells by flow cytometry. Briefly, the cells were cultured and treated with Y-27632 (5, 10 and 20 µM). After 10 days, the colonies were collected and by using 0.25% trypsin-EDTA and vortexing the single cells harvested. The cells were then washed with cold PBS, adjusted to 1×10^6^ cells/ml in 1X binding buffer and stained with Annexin V-FITC and PI solution for 15 min at room temperature. Stained cells were immediately analyzed by flow cytometry (BD FACSCalibur), and the data were analyzed using Cyflogic V.1.2.1 software.

### Intracellular ROS analysis

Total cellular ROS generation was detected with 20 µM 2′, 7′-dichlorodihydrofluorescein diacetate (ab113851, Abcam) using an FC500 MCL (Beckman Coulter), with excitation and emission wavelengths of 488 nm and 530 nm, respectively. According to the manufacturer’s instructions, 5 µM MitoSOX was added to the plates, followed by incubation for 10 min at 37 °C. The cells were then washed twice with pre-warmed (37 °C) HBSS Ca/Mg and detached with 0.25% trypsin. Fluorescence was measured using a FACSCalibur (BD Biosciences), with excitation and emission wavelengths of 488 nm and 580 nm. The data were analyzed with FlowJo software (FlowJo, LLC), version 10.

### Statistical analysis

The data from viability, colony formation, mean colony area, apoptosis, necrosis, ROS and real-time-PCR analysis of *Plzf*, *Cmyc*, *Vasa*, *Thy1*, *Bcl-2*, *Bax* and *p53* genes of SSCs under different concentrations of Y-27632 were analyzed with a statistical software program (SPSS 16, IBM, USA, 2007). Normality was checked using the Shapiro-Wilk test. One-way ANOVA followed by Duncan multiple-range test were used for making comparisons between multiple numeric datasets. Three biological and three technical replicates were used. The results were expressed as mean±SEM and statistical significance was accepted at P<0.05.

## Results

### Viability of SSCs after 48h culture

**Figure 1:**
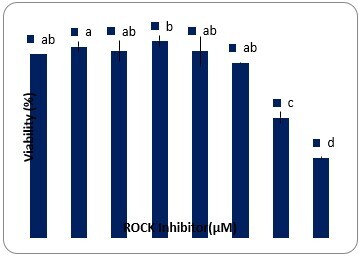
The effects of different concentrations of Y-27632 (0.1- 40 µM) on the viability of SSCs after 48h treatment (Control= 0 µM Y-27632). The data refer to the mean ± SEM. Different letters (a-d) on the bars indicate statistical difference (P < 0.05) by the Duncan test.

As shown in Figure 1, the viability of SSCs was not affected by 0.1 -10 µM Y-27632; however, higher concentration of Y-27632 (20 and 40 µM) significantly decreased the viability of SSCs (P<0.05).

### SSCs colony formation

**Figure 2:**
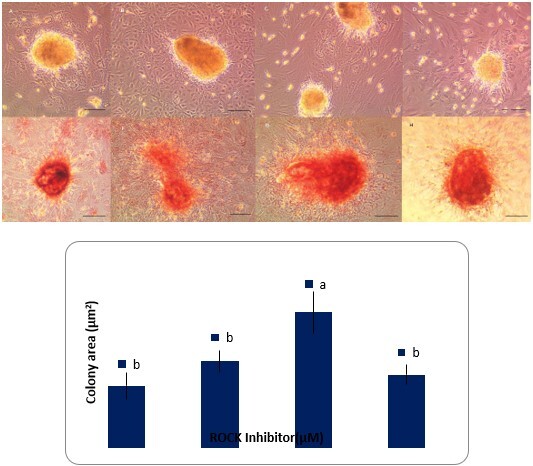
Ovine SSCs colonies, Alkaline phosphatase (AP) staining and mean colony area in cell cultures supplemented with different concentrations of Y-27632 after a 10-day culture (Control= 0 µM Y-27632). Y-27632 doses are 0 µM (A, E), 5 µM (B, F), 10 µM (C, G), and 20 µM (D, H). The data refer to the mean ± SEM. Different letters (a, b) on the bars indicate statistical difference (P < 0.05) by the Duncan test. Scale bars 50 µm.

**Figure 3:**
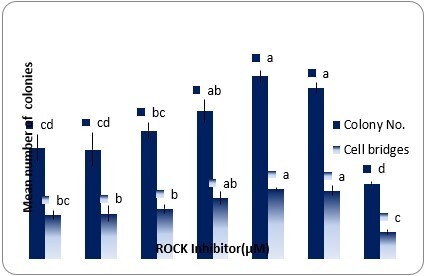
The effects of different concentrations of Y-27632 (0.1- 20 µM) on SSCs colony formation after 10 days (Control= 0 µM Y-27632). The data refer to the mean ± SEM. Different letters (a-d) on the bars indicate statistical difference (P < 0.05) by the Duncan test.

To clarify the effect of Y-27632 on ovine SSCs colony formation, different concentrations of Y-27632 were added to the SSCs cultures. Germ cells cultured in the media containing 0, 5, 10 and 20 µM of Y-27632 showed AP activity regardless of the Y-27632 dose. The mean area per AP positive colonies after 10 days of primary culture was significantly greater in the 10 µM Y-27632 group compared with the other groups (P<0.05) (Figure 2). As shown in Figure 3, with an increase in the Y-27632 dose from 0 to 10 µM, the means of colonies and cell bridges increased in a dose-dependent manner and they were significantly higher in the 5 and 10 µM Y-27632 groups than the control group (P<0.05). However, with the addition of 20 µM Y-27632, the means of colonies and cell bridges were reduced. Based on these results, it can be assumed that the data support beneficial effects of 10 µM Y-27632 on formation of SSCs colonies.

### Apoptosis assay of SSCs after a 10-day culture

**Figure 4:**
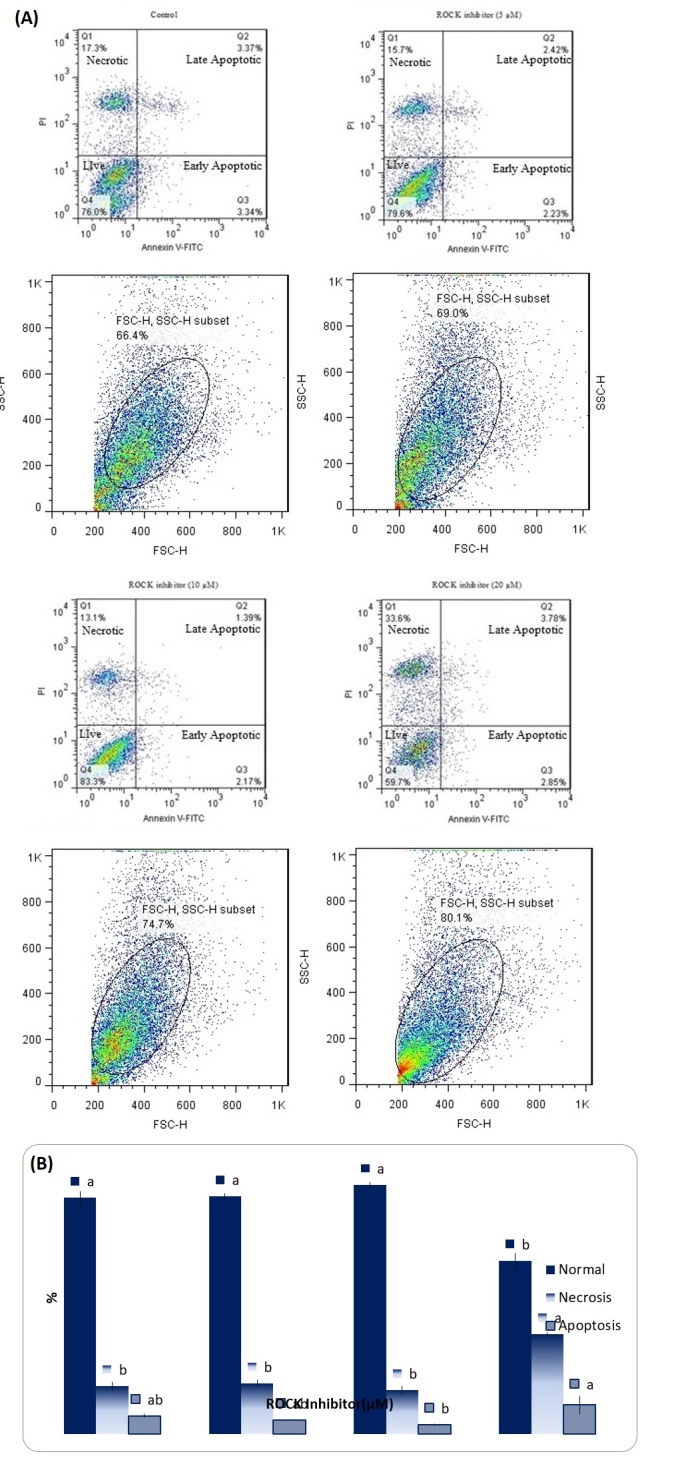
(A) Annexin-V/-FITC/PI Flow cytometry analysis of SSCs treated with 5, 10 and 20 mM of Y-27632 after a 10-day culture (Control= 0 µM Y-27632). The viable cells, early apoptotic and necrotic/secondary necrotic cells was represented by the lower left quadrant (Annexin-V^-^/PI^-^), lower right (Annexin-V^+^/PI^-^) and upper (Annexin-V^+^/PI^+^) quadrant, respectively. (B) Percentage of normal, necrosis and apoptosis after Y-27632 administration in SSCs cells. The data refer to the mean ± SEM. Different letters (a, b) on the bars indicate statistical difference (P < 0.05) by the Duncan test.

The flow cytometry results of SSCs after a 10-day culture with different concentrations of Y-27632 on cell viability, apoptosis, and necrosis are shown in Figure 4. The results demonstrated that 5 and 10 µM of Y-27632 did not significantly affect the cell viability, apoptosis, and necrosis of SSCs in comparison with the control group (P>0.05). However, 20 µM of Y-27632 significantly decreased the cell viability and increased the necrosis of the cells under study (P<0.05).

### Intracellular ROS analysis

**Figure 5:**
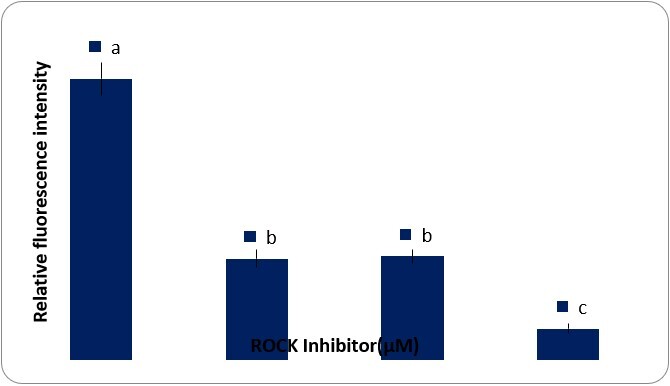
Analysis of intracellular reactive oxygen species (ROS) levels in the media supplemented with various concentrations of Y-27632 (Control= 0 µM Y-27632). The data refer to the mean ± SEM. Different letters (a-c) on the bars indicate statistical difference (P < 0.05) by the Duncan test.

The relative intracellular ROS levels were estimated 10 days after culture. As shown in Figure 5, the levels of intracellular ROS significantly decreased with an increase in the Y-27632 dose from 5 to 20 µM, and the ROS level was the lowest in the 20 µM group in contrast with other groups (P<0.05). However, no significant difference in ROS levels was observed between the 5 and 10 µM groups (P>0.05).

### SSCs markers gene expression

**Figure 6:**
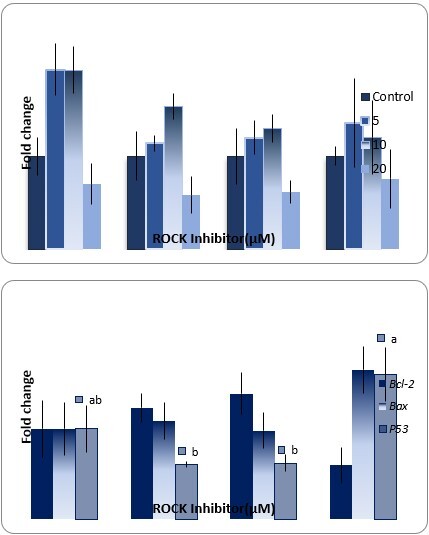
Real-time-PCR analysis of *Plzf*, *Cmyc*, *Vasa* and *Thy1* genes and *Bcl-2*, *Bax* and *p53* genes on day 10 after culture (C= 0 µM Y-27632). The data refer to the mean ± SEM. Different letters (a, b) on the bars indicate statistical difference (P < 0.05) by the Duncan test.

As shown in Figure 6, on day 10 after culture, there was no significant difference in the expression of *Plzf*, *Cmyc*, *Vasa* and *Thy1* genes among different concentrations of Y-27632 (0 to 20 µM).

(P>0.05).

### Apoptosis related gene expression

As shown in Figure 6, on day 10 after culture, *p53* expression decreased with an increase in the Y-27632 dose from 0 to 10 µM. However, in 20 µM Y-27632, the expression of *p53* significantly increased (P<0.05). In contrast*,* the relative mRNA levels of *Bcl-2* was the lowest in 20 µM Y-27632. Also, no significant difference in expression of *Bcl-2 and Bax* was observed in different concentrations of Y-27632 (0-20 µM) (P>0.05).

## Discussion

Establishing SSCs culture is usually a challenging matter due to the reduction in cell viability and proliferation. Modifications in the original cell features can be considered as another reason, which makes this process very challenging. Initially, there were some attempts to enhance SSCs viability through adding growth factors in the culture medium. However, these attempts did not lead to much improvement (Pramod & Mitra, 2014). ROCK inhibitors like Fasudil, Y-27632, Y39983, Wf-536, and SLx-2119 have been recently used in order to improve cell proliferation rate (Liao et al., 2007). As to the inhibitors mentioned above, Y-27632 was widely employed for pluripotent or adult stem cells in order to enhance their survival and proliferation rate and increase the cryopreservation efficiency (Lee et al., 2015). In other cases, Y-27632 was employed in order to improve the seeding efficiency of hiPSCs selected from transfected fibroblast cultures for primary colony development (Kurosawa, 2012). However, to the best of the researchers’ knowledge, no study has been performed to determine whether Y-27632 also improves the primary culture of SSCs.

Morphologic analyses indicated that the 5-10 µM Y-27632 had an optimal effect on the number of presumptive SSCs colonies and the area covered by them after a 10-day culture. According to the study conducted by Nam et al. (2011), cell attachment was accommodated by Y-27632 after dissociation and the ensuing survival. According to the results obtained in their research, there might be an enhancement in cell-cell and cell-extracellular matrix interactions after Y-27632 treatment. In another study, Lee et al. (2015) showed that Y-27632 influenced the maintenance of stem cell when the subculture was transferred through suppressing the expression of senescence-related proteins and increasing the cellular proliferation (Bi et al., 2013). Furthermore, Koga et al. (2006) realized that Y-27632 had the potential to improve cellular adhesion in other cell types. For instance, the addition of Y-27632 improved the adhesion of human trabecular meshwork cells to fibronectin or collagen type I.

In this study, no significant difference was observed in the expression SSCs markers (*Plzf*, *Cmyc*, *Vasa* and *Thy1)* among different concentration of Y-27632 (0 to 20 µM). The expression of *Vasa* is specific for the germ cell lineage (Abbasi et al., 2013). *Vasa* and *Plzf* are specific to cultured spermatogonia characterization (Wang et al., 2014) and their expressions are higher in SSCs than in somatic feeder cells (Azizi et al., 2019). It has been reported that *Plzf* is expressed in identical spermatogonia and served as particular regulator in order to promote the stem cell self-renewal in the testis (Costoya et al., 2004; Kubota et al., 2004; Oatley et al., 2006). The role of *Plzf* in spermatogonia is the maintenance of an undifferentiated state and self-renewal (Zheng et al., 2014; Qasemi-Panahi et al., 2018), which resembles the role proposed for *Plzf* in haematopoietic precursor cells (Reid et al., 1995). Borjigin et al. (2010) realized that *Plzf* plays the role of a molecular marker of sheep SSCs. Furthermore, having conducted practical transplantation studies on rodent testes and goat SSCs colonies, Wang et al. (2014) confirmed that *Thy1* would be the most exclusive marker of SSCs conducted on phenotypic characteristics and/or genotypic signatures. These features and signatures included *Thy1* and *plzf*, which are specially expressed in testes, proliferating identical SSCs of medaka (Oryzias latipes) (Hong et al., 2011), dogfish (Bosseboeuf et al., 2013), and rohu carp (an economically significant farmed carp) (Mohapatra et al., 2010; Panda et al., 2011).

Results showed that with an increase in the Y-27632 dose from 0 to 10 µM*,* in contrast to *p53* and *Bax,* the relative mRNA levels of *Bcl-2* increased and its expressions was the lowest in 20 µM Y-27632. *Bcl-2* is a strong anti-apoptotic molecule. This molecule was identified for the first time in follicular B-cell lymphoma as a translocation to the immunoglobulin heavy chain Locus-T, which causes the gene to be constitutively hyper-expressed (Tsujimoto et al., 1985). *Bax* is identified as apoptosis effectors (Echeverry et al., 2013). The *Bax* family is concerned as further downstream with mitochondrial disorder. *Bax* plays a significant role in releasing apoptogenic proteins, such as cytochrome c, by mediating mitochondrial membrane channels. Moreover, non-*Bcl-2* family proteins, such as *p53*, have been involved in organizing mitochondrial outer membrane permeability (Cory & Adams, 2002).

It seems that Y-27632 improves the survival of hESCs through complementary mechanisms such as increasing cellular adhesion by advancing more potent cell-cell interaction (Li et al., 2009). Although Li et al. (2009) showed the role of Y-27632 in decreasing the level of apoptosis, it is well understood that the ROCK pathway is closely related to the induction of cell apoptosis (Hwang et al., 2013). In comparison with the control group, the level of intracellular ROS decreased with an increase in the Y-27632 dose from 5 to 20 µM. Even though high levels of ROS might result in cellular injury and apoptosis because of damages to the membrane, lysis of cells, organelles dysfunction, and calcium dyshomeostasis (Kushki et al., 2015), low concentrations of ROS are fundamental to cells’ function such as the acquisition of fertilizing capability (Silva et al., 2010). Consequently, maintaining suitable ROS levels in SSCs culture media is very essential (Wang et al., 2014).

## Conclusion

In conclusion, the present study demonstrated that Y-27632 at a concentration of 5 -10 µM provided optimal culture conditions for the primary culture of SSCs, with normal expression of SSC-specific markers such as *Plzf*, *Cmyc*, *Vasa* and *Thy1* genes, up-regulation of *Bcl-2* as anti-apoptotic gene and down-regulation of *P53* as pro-apoptotic gene.
